# Sensitivity of different DNA extraction methods and PCR to detect resistance in patients with leprosy stratified by the bacilloscopic index

**DOI:** 10.1016/j.bjid.2022.102381

**Published:** 2022-06-27

**Authors:** Lais Sevilha-Santos, Danielle Costa Aquino, Günter Hans Neto, Fabiano José Queiroz Costa, Carlos Augusto Felipe de Sousa, Elaine Faria Morelo, Agenor de Castro Moreira dos Santos Júnior, Ciro Martins Gomes

**Affiliations:** a Faculdade de Medicina, Programa de Pós-graduação em Ciências Médicas, Universidade de Brasília, Brasília, DF, Brazil; b Universidade de Brasília, Hospital Universitário de Brasília, Brasília, DF, Brazil; c Laboratório Central de Saúde Pública do Distrito Federal, Secretaria de Saúde do Distrito Federal, Brasília, DF, Brazil; d Núcleo de Medicina Tropical, Universidade de Brasília, Brasília, DF, Brazil

**Keywords:** Leprosy, Microbial sensitivity tests, Polymerase chain reaction, Therapeutics, Bacilloscopic index, DNA extraction

## Abstract

**Introduction:**

Antimicrobial resistance in leprosy is an emerging problem, and the quantitative impact of low bacilloscopic indexes (BIs) on the sensitivity of molecular tests is unknown. We aimed to evaluate the sensitivity of gene sequencing for the detection of mutations related to antimicrobial resistance in *Mycobacterium leprae* in patients with low BIs using an analytical model.

**Methods:**

Patients with leprosy were included and divided into two groups depending on their BIs (≥ 2+ and < 2+). The sensitivities of the two DNA extraction methods were compared after amplifying and sequencing the repetitive element (RLEP), folP1, rpoB and gyrA in *M. leprae*.

**Results:**

We included 56 patients with leprosy: 35 had BIs less than 2+ (22 had negative slit-skin smear [SSS] results) and 21 patients had BIs greater than or equal to 2+. The sensitivity of the amplification of the RLEP target and the gene sequencing of folP1, rpoB and gyrA was 50 to 70% lower in patients with a BI less than 2+ and was significantly reduced in patients with lower BIs for all targets (*p* < 0.001). One patient had a mutation in the folP1 gene, and 14 patients had mutations in the gyrA gene, but no mutations related to antimicrobial resistance were found.

**Conclusions:**

We can conclude that the sensitivity of molecular tests is directly related to the BI, but these tests can still detect up to 20% of the targets in patients with BIs < 2+. New strategies to improve the sensitivity for detecting antimicrobial resistance in leprosy patients and reasonable clinical criteria for follow-up and the introduction of alternative treatments must be developed.

## Introduction

Leprosy is a neglected disease caused by *Mycobacterium leprae*, which is the second most common human pathogen of that genus, and by *Mycobacterium lepromatosis*.^1^^,^^2^ The disease is characterized by a chronic course and neurological sequelae and disabilities. Early treatment is the most important method for preventing deformities and the most effective method for breaking the chain of transmission.^2^

Similar to other mycobacteria, *M. leprae* is relatively resistant to most existing antimicrobials. The multi-drug therapy recommended by the World Health Organization (WHO) consists of the use of rifampicin, clofazimine and dapsone for six to 12 months, depending on the clinical presentation.^3^ Recent evidence supports the effectiveness of this treatment for curing leprosy.^4^ However, alternative treatments must be used by some patients with treatment intolerance, adherence problems and infection with resistant strains.^5^, ^6^, ^7^ Some drugs, including minocycline, ofloxacin, and clarithromycin, are interesting alternatives.^4^^,^^8^ Although *M. leprae* is still not culturable *in vitro,* vigilance and study of antimicrobial resistance are important.^9^ For many years, the *in vivo* mouse footpad inoculation method described by Shepard was the only reliable technique.^4^^,^^10^ Currently, the detection of gene mutations rather than the observation of clinical and laboratory signs of resistance is the most important strategy for the detection of infections with resistant strains of *M. leprae* because of its cost-effectiveness.^4^

The detection of mutations that lead to antimicrobial resistance in *M. leprae* depends on accurate laboratory procedures, including DNA extraction. The viability of the DNA and success of the amplification step also depend on the acquisition of an adequate amount of genetic material. This finding explains why some guidelines and studies recommend performing molecular tests for resistance in patients with leprosy presenting a bacilloscopic index (BI) greater than 2+.^9^ Although we might expect that patients who experience therapeutic failure due to infections with resistant *M. leprae* strains would also have a bacillary load that would not decrease over time, we must assume that some patients will experience intense but incomplete bacillary clearance. Some leprosy cases caused by resistant strains possibly result in a transitorily undetectable BI, although the disease remains active, especially in tissues into which the penetration of drugs is suboptimal.^11^ This explains why all patients, including patients with relatively low BIs, need to undergo investigations for antimicrobial resistance if it is clinically suspected. The quantitative impact of a low BI on the sensitivity of molecular tests with regard to the detection of antimicrobial resistance in patients with leprosy is still unknown.

We evaluated the sensitivity of the sequencing of the folP1, rpoB and gyrA genes to detect mutations related to antimicrobial resistance in *M. leprae* in patients with low BIs using an analytical model. We also tested the effects of the use of different laboratory procedures on the sensitivity of the detection of the DNA targets in *M. leprae*.

## Materials and methods

### Recruitment

Our target population was defined as local patients included in the Brazilian system for the surveillance of primary and secondary antimicrobial resistance in leprosy.^12^ This system selects all patients suspected of having leprosy relapses and at least 10% of those with new-onset leprosy for testing according to the WHO recommendations.^9^ Patients were recruited at Hospital Universitario de Brasília, Brazil, a specialized ambulatory unit for the diagnosis and treatment of patients with leprosy. Patients with leprosy before or after up to three months of treatment were consecutively included from August 2018 to September 2019. Laboratory exams were performed at the Dermatomycology Laboratory – Universidade de Brasília and at the Central Public Health Laboratory – LACEN, Distrito Federal, Brasília. Patients who did not sign the informed consent form were excluded. After inclusion, patients were divided into two groups according to their BIs (≥ 2+ and < 2+). The BI was calculated using the method described by Ridley in 1962 and was based on a logarithmic scale ranging from 0 to 6.^13^ The patient's BI was calculated by determining the arithmetic mean of the BIs for each analyzed site. The slit skin smear (SSS) was collected at the same time of PCR testing according to the method proposed by the Brazilian Vigilance System. Patients were classified prospectively.^12^

### Sample collection and DNA extraction

A 4-mm incisional biopsy was collected by the same board-certified dermatologist using an antiseptic protocol and local anesthesia with a 2% lidocaine solution. The site from which the sample was taken was the border of a skin lesion or infiltration. When no lesion was detected, a biopsy was taken from the back of the right earlobe.

The collected skin fragment was divided vertically into two fragments to test two different commercial DNA extraction kits: PureLink Genomic DNA Mini Kit (Invitrogen, Thermo Fisher Scientific, Waltham, Massachusetts, USA) and NucleoSpin Tissue XS (Macherey-Nagel, GmbH & Co. KG, Düren, Germany). Both kits were used according to the manufacturer's instructions.

### Polymerase chain reaction for M. leprae

For both extracted DNA samples, polymerase chain reaction (PCR) assays were performed using primers targeting the repetitive element (RLEP) region of *M. leprae*. The primer pair selected for this study resulted in a 148-base pair product (Table 1).^14^^,^^15^ Reactions were performed in a final volume of 30 µL containing 1x reaction buffer, 0.2 mM dNTPs, 1.5 mM MgCl, 1 U Platinum Taq DNA Polymerase (Invitrogen, Waltham, USA), 0.2 μM of each primer (Invitrogen, Waltham, USA), ultrapure water and 50–100 ng of genomic DNA. Amplification was performed with a T100 Thermal Cycler (Bio Rad, Hercules, USA) with an initial denaturation period of 3 min at 94°C followed by 15 cycles of 94°C for 30 s, 56°C for 30 s, and 72°C for 30 s, followed by 20 cycles of 94°C for 1 min, 56°C for 30 s, and 76°C for 1 min.

**Table 1:** Primer pairs used for polymerase chain reaction.

Target	Primer names	Sequences	Product length	GC%	Tm
RLEP	RLEP-F RLEP-R	5`-TGCGCTAGAAGGTTGCCGTAT-3` 5`-ATTTCTGCCGCTGGTATCGGT-3`	148	52.38 52.38	62.17 62.19
folP1	folP1-F1 folP1R1	5` - CTTGATCCTGACGATGCTGT - 3` 5` - CCACCAGACACATCGTTGAC - 3`	254	50.00 50.00	57.69 58.85
folP1	folP1-F2 folP1-R2	5` - GATCCTGACGATGCTGTCCAG - 3` 5` - ACATCGTTGACGATCCGTG - 3`	242	57.14 52.63	60.54 57.97
rpoB	rpoB-F1 rpoB-R1	5` - ACGCTGATCAATTATCCGTCC - 3` 5` - GTATTCGATCTCGTCGCTGA - 3`	345	47.62 50.00	58.24 57.33
rpoB	rpoB-F2 rpoB-R2	5` - CTGATCAATATCCGTCCGGT - 3` 5` - CGACAATGAACCGATCAGAC - 3`	255	50.00 50.00	56.89 56.65
gyrA	gyrA-F1 gyrA-R1	5` - ATGACTGATATCACGCTGCCA - 3` 5` - ATAACGCATCGCTGCCGGTGG - 3`	390	47.62 61.90	59.59 65.97
gyrA	gyrA-F2 gyrA-R2	5`- GATGGTCTCAAACCGGTACATC - 3` 5` - ACCCGGCGAATTGAAATTG - 3`	225	50.00 47.37	58.80 56.89

RLEP, Repetitive element; folP1, dapsone resistance-associated target; rpoB, rifampicin resistance-associated target; gyrA, quinolone resistance-associated target.

### Nested polymerase chain reaction for the folP1, rpoB and gyrA genes

When both kits resulted in amplification of the RLEP, we performed a nested PCR to detect resistance mutations using samples of DNA extracted with the PureLink Genomic DNA Mini Kit. The primers selected for the amplification of the folP1 (dapsone), rpoB (rifampicin) and gyrA (quinolones) genes are described in Table 1. The PCR program consisted of one hold cycle at 94° C for 2 min; followed by 30 cycles 94° C for 30 s, 56° C for 30 s, and 72° C for 30 s; and a final hold cycle at 72°C for 5 min.^16^ The PCR products of all reactions were visualized with a 2% agarose gel stained with GelRed (Biotium, Fremont, USA) and then purified for further sequencing using NucleoSpin Gel and PCR Clean-up (Macherey-Nagel, GmbH & Co. KG, Düren, Germany) following the manufacturer's instructions.

### Gene sequencing

The sequencing of folP1, rpoB and gyrA was performed using the amplicons obtained from the nested PCR. Therefore, 5 μL of PCR product was purified with ExoSAP-IT PCR Product Cleanup Reagent (Thermo Fisher Scientific, Waltham, EUA) at 37°C for 5 min. For each gene, a sequencing reaction was prepared using 3 μL of purified PCR product, 0.3 μM primer and the BigDye Terminator v3.1 Cycle Sequencing Kit (Life Technologies, Carlsbad, California, United States) following the manufacturer's instructions. Sequencing analyses were performed on an ABI 3500 Genetic Analyzer (Life Technologies, Carlsbad, California, United States). The obtained sequences were analyzed using Sequencher Alignment Editor Software v. 4.1.4. (Gene Codes Corporation, Ann Arbor, USA) and compared with known sequences in GenBank (National Center for Biotechnology Information, USA).

### Evaluation of samples and statistical analysis

We evaluated all the data of the target population for one year. Test sensitivity was evaluated based on a post-hoc analysis. Demographic characteristics were compared using the chi-squared test or Fisher's exact test. The mean numerical values in each group were compared using Student's t-tests. The sensitivity was defined as the number of positive test results among all included patients with leprosy. In the statistical analysis of the results stratified according to the biopsy collection site, results were adjusted based on the BI using a logistic regression model. All analyses were performed in RStudio software (Integrated Development Environment for R. RStudio, PBC, Boston, MA URL http://www.rstudio.com/). Significant values were defined by *p* < 0.05 and are reported with the corresponding 95% confidence intervals (CI).

### Ethics

The present research complied with the principles of the Declaration of Helsinki and was approved by the Ethics Committee of the Faculty of Medicine, Universidade de Brasília, Brazil (CAAE: 93119018.7.0000.5558). All patients were included after signing an informed consent form.

## Results

Fifty-six patients with leprosy were included in the study: 35 with BIs less than 2+ as evaluated using SSS (22 with negative SSS results) and 21 patients with BIs greater than or equal to 2+. Both groups were similar with regard to demographic characteristics, including sex, age and a previous history of leprosy treatment (Table 2). The proportion of patients experiencing leprosy reactions was greater in the higher BI group, and patients with a high BI were more likely to experience type II leprosy reactions.

**Table 2:** Demographic characteristics and comparisons between the groups stratified by bacilloscopic index.

	Bacilloscopic Index			
Variable	≥ 2 (*n* = 21)	< 2 (*n* = 35)	Total	*p*-value
**Sex**				
M, n (%)	17 (80.95%)	20 (57.14%)	37 (66.07%)	0.086
F, n (%)	4 (19.05%)	15 (42.86%)	19 (33.93%)	
Age: mean (SD)	43.24 (14.68)	44.31 (15.90)	43.91 (15.32)	0.802
Previous treatment	11 (52.38%)	15 (42.86%)	25 (44.64%)	0.678
**Reactions**				0.001
Type I	5 (23.81%)	15 (42.86%)	20 (35.71%)	
Type II	5 (23.81%)	1 (2.86%)	6 (10.71%)	
Type I and II	9 (42.86)	5 (14.29%)	14 (25.00%)	
None	2 (9.52%)	14 (40.00%)	16 (28.57%)	

*n*, number of patients; SD, standard deviation.

The operational classification, the Madrid classification and the Ridley & Joplin classification are shown in Table 3. As expected, patients with a higher BI were more frequently classified as having lepromatous-lepromatous leprosy, indicating that the clinicians likely applied the classification criteria appropriately. The BI was neither related to the type of leprosy treatment prescribed nor to the prescription of any alternative treatment, probably because the research center is a reference facility that prioritizes patients with advanced infections, including refractory reactions (Table 4).

**Table 3:** Comparison of leprosy classifications between the groups stratified by bacilloscopic index.

	Bacilloscopic Index		
Classification	≥ 2 (n = 21)	< 2⁎ (n = 35)	*p*-value
**Operational**			
Paucibacillary	0	8	0.020
Multibacillary	21	27	
**Madrid**			
Indeterminate	0	2	0.001
Tuberculoid	0	9	
Borderline	6	15	
Lepromatous	15	9	
**Ridley & Joplin**			
Indeterminate	0	2	0.001
Tuberculoid-Tuberculoid	0	4	
Tuberculoid-Borderline	0	5	
Borderline-Borderline	3	14	
Borderline-Lepromatous	3	2	
Lepromatous-Lepromatous	15	8	

⁎ Including negative slit skin smears.

**Table 4:** Differences in prescribed treatments between the groups stratified by bacilloscopic index.

	Bacilloscopic Index			
Variable	≥ 2 (*n* = 21)	< 2 (*n* = 35)	Total	*p*-value
Previous treatment	11 (52.38%)	15 (42.86%)	25 (44.64%)	0.678
Alternative treatment⁎	11 (52.38%)	16 (45.71%)	27 (48.21%)	0.136
ROM	2 (9.52%)	0	2 (3.57%)	
WHO MB-MDT	8 (38.10%)	19 (54.29%)	27 (48.21%)	
Rifampicin	20 (95.24%)	34 (97.14%)	54 (96.43%)	1
Dapsone	14 (66.67%)	25 (71.43%)	39 (69.64%)	0.940
Clofazimine	21 (100%)	34 (97.14%)	55 (98.21%)	1
Ofloxacin	12 (57.14%)	16(45.71%)	38 (67.86%)	0.581
Minocycline	7 (33.33%)	6 (17.14%)	13 (23,21%)	0.288
Moxifloxacin	2 (9.52%)	6 (17.14%)	8 (14.29%)	0.696

ROM,monthly rifampicin + daily ofloxacin and minocycline; WHO MB-MDT, World Health Organization Multibacillary Multidrug Therapy.

⁎ Any treatment different from regular World Health Organization Multidrug Therapy.

The sensitivity of conventional PCR for the amplification of the RLEP, folP1, rpoB and gyrA was 50 to 70% lower in patients with a BI less than 2+ (Table 5). The sensitivity was significantly lower in patients with a lower BI for all targets (*p* < 0.001). Both extraction kits yielded a similar sensitivity for the detection of *M. leprae* independent of the BI *(*McNemar's *p*-value = 0.628), although the concordance between the two tests was not satisfactory (Kappa = 0.38; 95% CI = 0.12-0.64; *p* = 0.002). In patients with a negative SSS, the PureLink Genomic DNA Mini Kit seems to result in a more sensitive detection of the RLEP (sensitivity = 40.90%; 95% CI = 23.26-61.27) than the NucleoSpin XS kit (sensitivity = 31.81%; 95% CI = 16.36-52.68). This difference was not significant (McNemar's *p*-value = 0.505); the Kappa statistic was also not satisfactory for this comparison (Kappa = 0.15; 95% CI = 0.20-0.75; *p* = 0.450), meaning that the two extraction techniques may have complementary properties. Our analytical approach showed no relationship between treatment time (up to three months) and sensitivity results (*p* > 0.05). We also did not observe a relationship between the biopsy site (lesion or earlobes of patients without cutaneous lesions) and sensitivity, even when the results were adjusted for the BI (Table 6).

**Table 5:** Sensitivity and 95% CIs of diagnostic techniques and resistance detection in the groups stratified by the BI.

	Bacilloscopic Index				
Test	≥ 2 (*n* = 21)	< 2 (*n* = 35)	0+(Negative BI)	Total	*p*-value
**PCR RLEP**					
PureLink Kit	19 (90.48%) (71.09-97.35)	15 (42.86%) (27.98-59.14)	9 (40.90%) (23.26-61.27)	34 (60.71%) (47.63-72.42)	<0.001
Nucleospin TXS	19 (90.48%) (71.09-97.35)	12 (34.29%) (20.83-50.85)	7 (31.81%) (16.36-52.68)	31 (55.36%) (42.41-67.61)	<0.001
Complementary sensitivity*					
folP1	19 (90.48%) (71.09-97.35)	8 (22.86%) (12.07-39.02)	5 (22.73%) (10.12-43.44)	27 (48.21%) (35.67-60.99)	<0.001
rpoB	19 (90.48%) (71.09-97.35)	7 (20.00%) (10.04-35.89)	3 (13.64%) (4.749-33.34)	26 (46.43%) (34.02-59.30)	<0.001
gyrA	16 (76.19%) (54.91-89.37)	5 (14.28%) (6.26-29.38)	2 (9.09%) (2.529-27.82)	21 (37.50%) (26.01-50.59)	<0.001

⁎ For sensitivity calculation, we considered a result positive if either of the extraction kits resulted in the amplification of the target genetic sequence. PureLink, PureLink Genomic DNA Mini Kit (Invitrogen, Thermo Fisher Scientific, Waltham, Massachusetts, USA); Nucleospin TXS, NucleoSpin Tissue XS (Macherey-Nagel, GmbH & Co. KG, Düren, Germany); folP1, dapsone resistance-associated target; rpoB, rifampicin resistance-associated target; gyrA, quinolone resistance-associated target.

**Table 6:** Sensitivity and 95% CIs of diagnostic techniques and resistance detection in the groups stratified by the biopsy collection site.

	Biopsy Collection Site			
Test	Earlobe (*n* = 23)	Lesion (*n* = 33)	*p*-value	Adjusted *p*-Value⁎⁎
**PCR RLEP**				
PureLink Kit	12 (52.17%) (32,96-70,76)	22 (66.67%) (49.61-80.25)	0.415	0.491
Nucleospin TXS	10 (43.48%) (25.63-63.19)	21 (63.64%) (46.62-77.81)	0.223	0.269
Complementary sensitivity*				
folP1	10 (43.48%) (25.36-63.19)	17 (51.52%) (35.22–67.50)	0.749	0.942
rpoB	9 (39.13%) (22.16-59.21)	17 (51.52%) (35.22-67.50)	0.521	0.795
gyrA	7 (30.43%) (15.60-50.87)	14 (42.42%) (27.24-59.19)	0.528	0.879

⁎ For the sensitivity calculation, we considered a result positive if either of the extraction kits resulted in the amplification of the target gene sequence.

⁎⁎ *p*-values were adjusted for BIs using a logistic regression model. PureLink, PureLink Genomic DNA Mini Kit (Invitrogen, Thermo Fisher Scientific, Waltham, Massachusetts, USA); Nucleospin TXS, NucleoSpin Tissue XS (Macherey-Nagel, GmbH & Co. KG, Düren, Germany); folP1, dapsone resistance-associated target; rpoB, rifampicin resistance-associated target; gyrA, quinolone resistance-associated target.

We observed a significant reduction in the sensitivity of the detection of folP1, rpoB and gyrA in patients with a BI less than 2+. This reduction in sensitivity was even greater in patients with negative SSS results. Nested PCR was capable of amplifying only 10 to 40% of the genetic targets in patients with a BI less than 2+. No mutations related to antimicrobial resistance were found in the analyzed samples. Only one patient had a substitution mutation in the folP1 gene (c.288G>A; p.Ala96=). We found no mutations in the rpoB gene. Fourteen patients had deletion-insertion mutations in the gyrA gene (c.352_353delinsAA; p.Gly118Asn), and 10 patients had a substitution mutation in the same gene (c.297C>T; p.Arg99=).

## Discussion

The emergence of antimicrobial-resistant strains of *M. leprae* is considered an ongoing public health threat. The WHO has made specific recommendations regarding the surveillance of antimicrobial resistance, which is a serious problem associated with many infectious diseases due to the inadequate investment of time and attention into the development of new drugs.^9^ Although a recent systematic review of the literature showed that the prevalence of antimicrobial resistance in *M. leprae* has not increased in the last decade, the fact that the diagnostic tests used to detect resistant strains are not perfect must be considered; surveillance must be performed continuously.^4^

This study identified a clear limitation of the tests used to detect antimicrobial resistance: such tests are not as useful in patients with low BIs. It is well known that the presence of PCR inhibitors and low DNA load can reduce the sensitivity of PCR. Other obstacles that can reduce the sensitivity of PCR include the occurrence of resistance mechanisms not related to DNA mutations and the occurrence of mutations not yet described in the literature. These limitations also, in part, hold true for the *in vivo* culturing of *M. leprae* because a low concentration of the bacteria will not yield satisfactory growth in animal models. These limitations do not suggest that patients with low BIs are not affected by resistant *M. leprae* strains. In fact, patients with partial resistance to one or more drugs or with simultaneous infections with resistant and sensitive *M. leprae* strains may achieve a significant reduction in their BIs with the WHO-recommended multidrug therapy but then develop late relapses after selection and replication of resistant strains.

New and more sensitive strategies for the detection of antimicrobial resistance in *M. leprae* must be developed and used for surveillance at the population level.^17^^,^^18^ Techniques such as real-time PCR are interesting alternatives.^17^^,^^19^ Real-time PCR followed by high-resolution melting curve analysis or the use of specific TaqMan probes probably yields more sensitive results than conventional PCR followed by gene sequencing.^4^ However, according to a recent systematic review of the literature, validation of those tests is still needed in well-designed accuracy studies.^4^

Although alternative strategies exist, no technique is likely to achieve 100% sensitivity in the detection of antimicrobial resistance in *M. leprae*. This fact indicates the need for clinical criteria to guide the selection of alternative treatments for suspected cases of resistance.^20^ Before initiating an alternative treatment, clinical providers must first thoroughly exclude the possibility of reinfection and ensure that adherence to the standard treatment was adequate.^21^^,^^22^ Therefore, repeated evaluation of household contacts and a detailed investigation of the patient's clinical history are essential before any alternative treatment is considered for patients with inconclusive tests for antimicrobial resistance. In suspected cases of clinical relapse with inconclusive antimicrobial resistance test results, no evidence of reinfection and adequate adherence to previous treatment, new clinical criteria guiding follow-up and the initiation of alternative treatments must be developed. The traditional criteria that were used before the introduction of polychemotherapy, such as skin lesion infiltration and the serial evaluation of the BI, may not be adequate if used alone because of their imprecise nature and the long time needed for those methods to show perceivable changes.

In the present population, a previously described resistance-related mutation was not identified. This result suggests that WHO multidrug therapy is still an important and cost-effective disease control measure. The early introduction of treatment is key to preventing the development of disabilities in affected patients and breaking the chain of transmission. Interestingly, although no resistance gene was found, a significantly greater number of mutations associated with quinolones were found in the gyrA gene. A potential explanation for this finding is that quinolones may be more prone to being affected by antimicrobial resistance than other drugs due to their more frequent use for common infections than rifampicin and dapsone.^23^^,^^24^ Recommendations regarding the appropriate prescription of fluoroquinolones and pharmacovigilance strategies need to be carefully made because this is a serious public health threat.

Some limitations of the present study must be taken into consideration when interpreting the results. Although the sensitivities were similar between the tested extraction kits, the concordance between the two techniques was unsatisfactory. This implies that the kits may have different properties and that they can be used as complementary techniques. However, we did not identify any clinical or laboratory evidence that could indicate when one extraction kit should be preferred over the other. Additionally, as mentioned above, additional causes of antimicrobial resistance may exist for which specific tests are unavailable.^4^

## Conclusions

We can conclude that tests for the diagnosis of antimicrobial resistance in leprosy may be 50 to 70% less sensitive in patients with BIs less than 2+ than in patients with higher BIs. However, those tests can still successfully detect the genetic targets in 10 to 20% of patients with low BIs. New strategies to improve the detection of antimicrobial resistance in patients with leprosy and reasonable clinical criteria for follow-up and the initiation of alternative treatments must be developed.

## Funding sources

This study was financed in part by the Coordenação de Aperfeiçoamento de Pessoal de Nível Superior - Brasil (CAPES) - Finance Code 001.

## CRediT authorship contribution statement

**Lais Sevilha-Santos:** Conceptualization, Methodology. **Danielle Costa Aquino:** Methodology. **Günter Hans Neto:** Investigation. **Fabiano José Queiroz Costa:** Investigation, Validation. **Carlos Augusto Felipe de Sousa:** Investigation, Validation. **Elaine Faria Morelo:** Writing – original draft. **Agenor de Castro Moreira dos Santos Júnior:** Writing – original draft. **Ciro Martins Gomes:** Writing – review & editing, Supervision.

## Conflicts of interest

The authors declare no conflicts of interest.
